# Fungal Diversity and Gibberellin Hormones Associated with Long Whips of Smut-Infected Sugarcanes

**DOI:** 10.3390/ijms25169129

**Published:** 2024-08-22

**Authors:** Syeda Wajeeha Gillani, Lixiu Teng, Abdullah Khan, Yuzhi Xu, Charles A. Powell, Muqing Zhang

**Affiliations:** 1 Guangxi Key Laboratory of Sugarcane Biology, State Key Laboratory for Conservation and Utilization of Subtropical Agro-Bioresources, Guangxi University, Nanning 530004, China; 1817401019@st.gxu.edu.cn (S.W.G.);; 2 College of Agriculture, Guangxi University, Nanning 530004, China; 3 Indian River Research and Education Center-Institute of Food and Agricultural Sciences (IRREC-IFAS), University of Florida, Fort Pierce, FL 34945, USA

**Keywords:** metagenomics, metabolomics, fungal community, *Sporisorium scitamineum*, *Candida*, *Fusarium*, differentially accumulated metabolites (DAMs)

## Abstract

Sugarcane smut, caused by the fungus *Sporisorium scitamineum* (Sydow), significantly affects sugarcane crops worldwide. Infected plants develop whip-like structures known as sori. Significant variations in these whip lengths are commonly observed, but the physiological and molecular differences causing these morphological differences remain poorly documented. To address this, we employed conventional microbe isolation, metagenomic, and metabolomic techniques to investigate smut-infected sugarcane stems and whips of varying lengths. Metagenomics analysis revealed a diverse fungal community in the sugarcane whips, with *Sporisorium* and *Fusarium* genera notably present (>1%) in long whips. Isolation techniques confirmed these findings. Ultra-performance liquid chromatography analysis (UHPLC-MS/MS) showed high levels of gibberellin hormones (GA_3_, GA_1_, GA_4_, GA_8_, and GA_7_) in long whips, with GA_4_ and GA_7_ found exclusively in long whips and stems. Among the prominent genera present within long whips, *Fusarium* was solely positively correlated with these gibberellin (GA) hormones, with the exception of GA_8_, which was positively correlated with *Sporisorium*. KEGG enrichment analysis linked these hormones to pathways like diterpenoid biosynthesis and plant hormone signal transduction. These findings suggest that *Fusarium* may influence GA production leading to whip elongation. Our study reveals fungal dynamics and gibberellin responses in sugarcane smut whips. Future research will explore the related molecular gibberellin synthesis mechanisms.

## 1. Introduction

Sugarcane (*Saccharum officinarum* L.) is a globally significant agricultural ratooning crop [1], responsible for 85% of the global sugar production and 40% of bio-energy production [2,3,4,5]. Sugarcane is cultivated on approximately 27 million hectares across 100 countries [3,6] with southern China, including Guangxi, Yunnan, and Guangdong Provinces, encompassing over 1.7 million hectares of sugarcane cultivation [7].

Sugarcane is susceptible to numerous pathogens during sprouting and ratooning, including *Sporisorium scitamineum* (Sydow) (syn. *Ustilago scitaminea* (Syd.)) which causes smut [8,9]. This fungus infects and reproduces within sugarcane’s meristematic tissues, forming long, black whip-shaped sori containing melanized teliospores (≥10^11^ spores/cm^2^) from the apical parts of infected stalks [8,9]. The symptoms become evident approximately 120 days after infection, leading to adverse effects such as reduced culm widths, abnormal grass-like growth, and highly fibrous stalks with decreased sugar concentration [10,11].

Reportedly, the impact of smut on meristem processes in sugarcane includes the up-regulation of genes linked to whip formation, substituting regular flowering, such as LNG (longifolia-like gene), VIN3 (vernalization insensitive 3 protein) homologs, COL6 (C2C2-CO- like transcription factor), FT (Flowering Locus T), AP1 (APETALA1), and the production of active gibberellins [12]. In a study, head smut (*Sporisorium reilianum* [Kuhn] Landon & Fullerton)-infected sorghum (*Sorghum bicolor* L.) plants showed reduced height, increased tillering, and lower GA levels, indicating that smut infection disrupted plants’ GA biosynthesis [13]. The same study also reported the quantification of GA1 and GA3 in the culture medium of *S. reilianum* using GC-MS-SIM [13]. Previous studies also reported that *S. reilianum* infection altered hormones in susceptible maize varieties, with reduced gibberellic acid (GA_3_) leading to dwarfing [14]. Plants, fungi, and bacteria all possess significant gibberellin (GA) concentrations, with commercial GA production relying on *Fusarium fujikuroi* (Nirenberg) due to its high yield [15]. *Fusarium fujikuroi* causes “bakanae” disease in rice, and results in elongated seedlings with chlorotic stems and leaves, leading to sterility and grain loss, and gibberellin production being a key factor in the elongation of infected plants [16,17]. While gibberellins in plants regulate plant growth, in species of *Fusarium*, such as *F. fujikuroi*, those are secondary metabolites presumed to enhance pathogen virulence [18]. The GA_3_ hormone was observed to promote internode lengthening and phyllody in sorghum plants, resembling a typical sign of sorghum head smut [19]. Additionally, GA_3_ isolated from a fungal medium had been reported to stimulate shoot elongation in sorghum [20]. Gibberellin-like substances produced by *Colletotrichum gloeosporioides* (Penz.) resulted in leaf and pod deformation and promoted host pathogenesis in sorghum [21]. Symbiotic endophytic fungi (*Penicillium* sp. and *Phoma glomerata* Corda.) producing GAs (GA_3_, GA_7_, GA_1_, and GA_4_) were reported to induce shoot elongation and increase chlorophyll content in cucumber (*Cucumis sativus* L.) plants exposed to abiotic stress [22]. Given that *Sporisorium* causes dwarfing, increases tillering, and alters GA levels in infected plants, it remains unclear whether *Sporisorium* alone is responsible for the variation in whip morphology or if other microbial communities and physiological factors contribute. Therefore, the roles of endophytic fungi and metabolites like gibberellins in smut whip growth require further investigation.

Our study aims to identify endophytic fungi influencing gibberellin production and smut-whip elongation in affected sugarcanes. Therefore, we conducted conventional isolation, microbiome sequencing, and ultra-performance liquid chromatography (UHPLC-MS/MS) analyses to: (i) characterize fungal diversity in smut-infected sugarcane whips and stems; (ii) quantify gibberellin levels in infected sugarcane samples; and (iii) determine the relationship between endophytic fungi and gibberellin production in whips, and its potential impact on whip elongation.

## 2. Results

Smut infection in sugarcane leads to phyllody and reduced plant height. A key characteristic of smut disease is the formation of whips, with significant variations in length, both exceptionally long and short. These variations are frequently observed during field surveys, suggesting underlying molecular or physiological changes driving these morphological differences. Consequently, this study focused on the possible role of endophytic fungi and gibberellins in causing smut-whip variation in the highly smut-susceptible sugarcane cultivar ROC22. We selected sugarcane stems with long (>100 cm) and short (<50 cm) smut whips, dried them in the shade, and separated them into four sample types: LW (long whips), SW (short whips), LS (long stalks corresponding to long whips), and SS (short stalks corresponding to short whips). Metagenomic and targeted metabolomic analyses were performed on six biological replicates per sample. Endophytic fungi were isolated using potato dextrose agar (PDA) as the growth medium, and fungal DNA was extracted for ITS region amplification and sequencing. High-throughput sequencing of metagenomic DNA was conducted on the Illumina HiSeq 2500 platform, and operational taxonomic units (OTUs) were identified to assess microbial diversity. Additionally, the GA levels in the sugarcane samples were quantified using Ultra-performance liquid chromatography–mass spectrometry (UHPLC-MS/MS).

### 2.1. Diversity of Fungal Communities Associated with Sugarcane Whip Lengths

**Figure 1:**
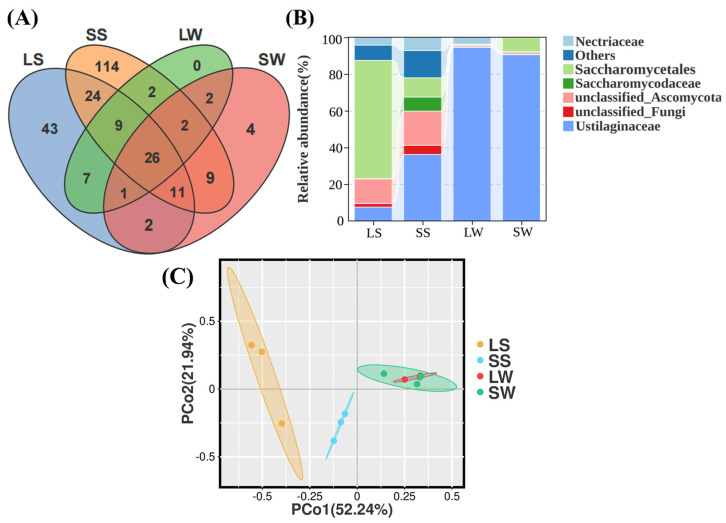
Venn diagram and community bar plot illustrating fungal community richness in different sugarcane samples. (**A**) Venn diagram of shared and unique OTUs among different stalk and whip samples. (**B**) Fungal community abundance (%) at the family level. LS: long stem, SS: short stem, LW: long whip, SW: short whip. (**C**) PCoA analysis at OTU level for smut-containing sugarcane shoot and whip samples of different lengths. LS: long stem, SS: short stem, LW: long whip, SW: short whip.

A total of 256 operational taxonomic units (OTUs) were identified (Table S1), with 26 OTUs present in all samples (Figure 1A).

Among these, 43 and 114 were unique to long (LS) and short (SS) stalk samples, respectively. In contrast, the long (LW) and short (SW) whip samples had 0 and 4 unique OTUs, respectively. These distinct OTUs provide valuable insights into the factors contributing to variations in whip- and stalk lengths. Additionally, the analysis of fungal diversity at the family level, represented in the community bar plot (Figure 1B), showed the predominant families and their richness in the studied samples. The endophytic fungal communities in four samples were precisely dominated by *Ustilaginaceae* (56.5%), *Saccharomycetales Incertae sedis* (19.5%), *Nectriaceae* (3.8%), *Saccharomycodaceae* (2%), *Rhynchogastremataceae* (1.6%), and *Tremellaceae* (1.1%) excluding unclassified families (Figure 1). Moreover, the relative abundance of *Ustilaginaceae* was 48% higher in whip samples (LW, 50%; SW, 45%) compared with stalk samples, while that of *Saccharomycetales Incertae sedis* was 21% lower (LW, 25%; SW, 16%). These specific taxa potentially contribute to variations in whip and stem lengths.

Alpha diversity indices were calculated to assess fungal diversity across samples (Table 1). Significant differences were observed between stem and whip samples. Short samples (SS, SW) exhibited higher Shannon and Chao indices compared with long samples (LS, LW), with SS having the highest values, except for the Simpson index (Table 1).

This indicated relatively minor differences in community richness between short and long whips, while short and long stems showed greater disparity, with SS showing the most varied diversity. Beta diversity, identified using PCoA analysis (R = 0.5957, *p* < 0.001), provided insights into fungal community structure similarities and differences across samples (Figure 1C). The PCoA plot accounted for 74.2% of the total variation, with PCo1 and PCo2 explaining 52.2% and 21.9%, respectively. Whip samples (LW, SW) were distinct from stem samples (LS, SS), and long and short whips (LW, SW) were closer to each other, indicating similar fungal community structures compared with the long and short stems (LS, SS). This analysis highlights the variations in community structure between sugarcane stem and whip samples (Figure 1C).

### 2.2. Dominant Fungal Genera Associated with Long Stem and Whip

**Figure 2:**
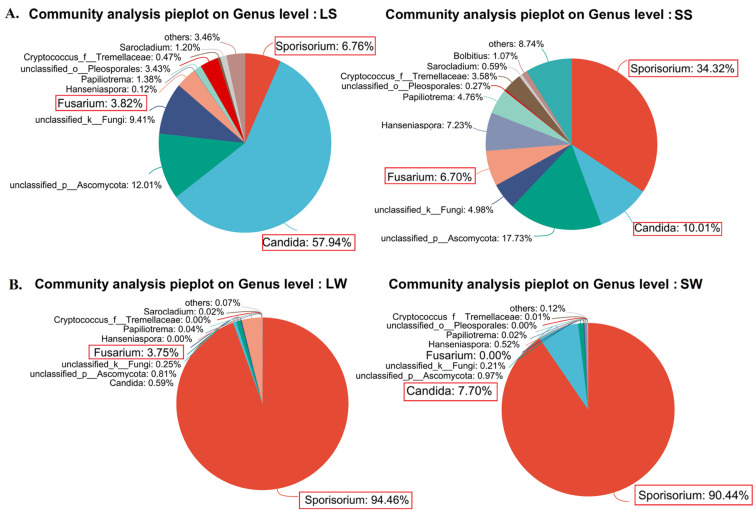
Pie plot of community composition displaying endophytic fungal genera in different sugarcane stem and whip samples. (**A**) Smut-induced sugarcane shoot samples. (**B**) Smut-induced sugarcane whip samples. LS: long stem, SS: short stem, LW: long whip, SW: short whip. The genera indicated by red boundaries are shared among samples.

In the long stem (LS) samples, the dominant genera (>3%) were *Candida*, *Sporisorium*, and *Fusarium*. In the short stem (SS) samples, the dominant genera were *Sporisorium*, *Candida*, *Hanseniaspora*, *Fusarium*, and *Papiliotrema*. Among the whip samples, the dominant genera (>1%) were *Sporisorium* and *Fusarium* in long whips (LWs), and *Sporisorium* and *Candida* in short whips (SWs) (Figure 2).

*Candida* was the most prevalent genus (>3%) in both stem samples (LS; SS), with an average abundance of 34.8%. In contrast, *Sporisorium* was prevalent in whip samples (LW; SW), with an average abundance of 92.4% (Figure 2). Moreover, the abundance of the genus *Candida* was 47.9% higher in long stems (LSs) compared with short stems (SSs), while the abundance of the genus *Fusarium*, after *Sporisorium* (smut pathogen), was 3.8% higher in long whips (LWs) compared with short whips (SWs) (Figure 2). Despite the percentage abundance, at the OTU level, *S. scitamineum* was the highest-ranked OTU cluster (>300,000 sequences), followed by *Candida jarooni* (Kurtzman & Robnett) with >85,000 sequences, and *Fusarium concentricum* (Nirenberg & O’Donnell) with >23,000 sequences across samples, excluding the unclassified species (Table S1). These representative sequences accurately reflect the abundant species within each cluster with over 97% nucleotide similarity. Therefore, these findings emphasize the significance of these genera in the morphological diversity and physiological fluctuations in these stems and whips.

These metagenomic results were further validated by conventionally isolated fungal sequences (Table 2, Figures S1 and S2). Abundant isolates of genera including *Sporisorium*, *Fusarium*, and *Sarocladium* were obtained from both long- and short-whip samples, with *F. verticillioides* (Sacc.) distinctly isolated from long (LS; LW) samples (Table 2).

### 2.3. Regulation of Gibberellin and Its Association with Fungi and Whip Lengths

Nine gibberellin hormones were detected and actively regulated in the four tested sample types (LS, SS, LW, SW), each revealing distinct quantitative changes (*p* ≤ 0.05) based on the source (Table 3).

**Figure 3:**
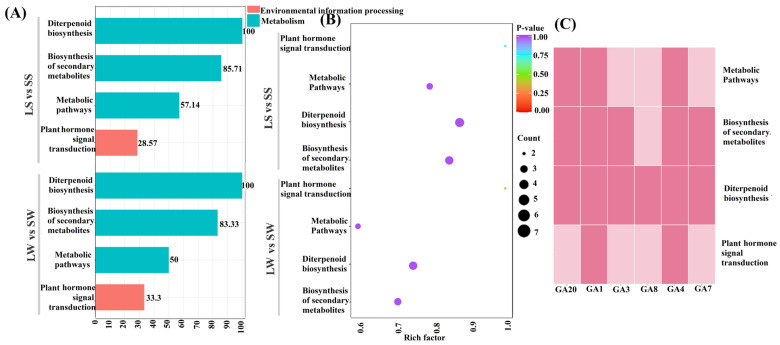
(**A**) KEGG annotation barplot. (**B**) KEGG enrichment scatter plot of differentially accumulated gibberellins (GAs) in LW vs. SW and LS vs. SS comparison analyses. Higher values indicate greater enrichment and redder points signify higher enrichment significance (*p* < 0.05). (**C**) Binary heatmap of differentially accumulated GAs across KEGG pathways. LS: Long stem; SS: Short stem; LW: Long whip; SW: Short whip; Ko: KEGG orthology.

Compared with short samples, the concentrations of five DAMs (GA_20_, GA_3_, GA_1_, GA_4_, GA_7_) were higher in the long samples. Notably, GA_3_ and GA_1_ were significantly higher in long-stem and long-whip samples (LS, LW), with the highest levels observed in long whips (Table 3). The GA_20_ content was highest in long stems (LS), while GA_4_ and GA_7_ were exclusively present in long samples (LS, LW), with the highest concentration in long whips (Table 3). To elucidate the physiological processes linked to these differentially accumulated metabolites (DAMs), Kyoto Encyclopedia of Genes and Genomes (KEGG) annotation and pathway enrichment analysis were performed for the comparison groups LS vs. SS and LW vs. SW. These analyses revealed that the differentially accumulated GAs in both groups were predominantly enriched in four pathways, with diterpenoid biosynthesis (ko00904) being the most prominent, followed by the biosynthesis of secondary metabolites (ko01110). Additionally, plant hormone signal transduction (ko04075) was more pronounced in LW vs. SW compared with LS vs. SS (Figure 3A,B).

**Figure 4:**
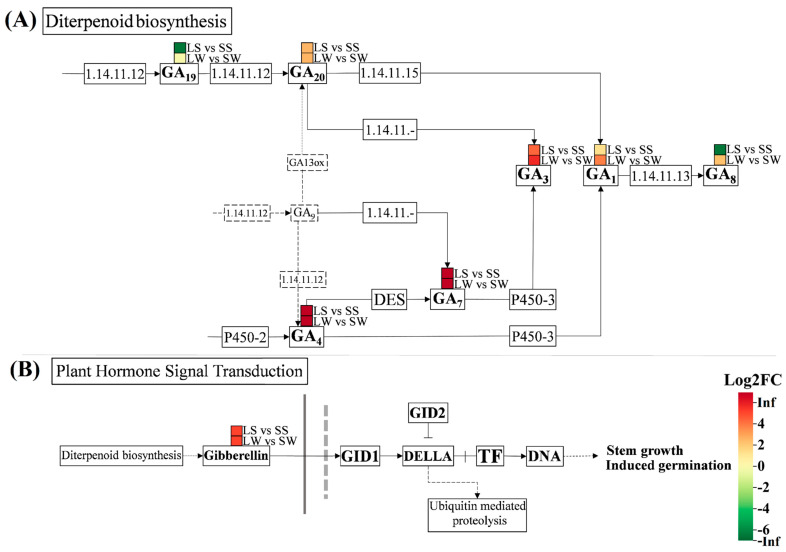
Schematic diagram of KEGG pathways associated with (**A**) diterpenoid biosynthesis (map00904), and (**B**) plant hormone signal transduction (map04075) between two comparison groups (LS vs. SS; LW vs. SW) shown by the colored cells. Positive Log2FC (fold change) values indicate GA upregulation, negative indicate downregulation, and zero signifies insignificant. DES: GA4 desaturase; TF: Phytochrome-interacting factor 4; LS: Long stem; SS: Short stem; LW: Long whip; SW: Short whip.

Among the detected DAMs, six were common to both comparison groups. GA_1_ and GA_4_ were annotated across all four pathways, whereas GA_8_ was exclusively linked to diterpenoid biosynthesis. Only two DAMs, GA_4_ and GA_1_, were annotated to the plant hormone signal transduction pathway (Figure 3C). Moreover, GA_19_ was found to be non-significant in LW vs. SW and was downregulated in the LS vs. SS comparison group. The regulatory trends of these DAMs are shown in Figure 4. Consequently, the diterpenoid biosynthesis and plant hormone signal transduction pathways were further examined. DAMs identified in both LS vs. SS and LW vs. SW comparisons revealed that all six common GAs were associated with the diterpenoid pathway. GA_8_ and GA_19_ were downregulated in long stems compared to short stems, and GA_19_ was non-significant in long whips compared with short whips (Figure 4).

These results also suggest that these physiological variations may be influenced by factors such as smut infection and the endophytic fungal community structure. The upregulation of gibberellin in long stems and whips during diterpenoid biosynthesis, as shown in Figure 4B, led to stem growth and germination. This process could contribute to the elongation of whip structures emerging from the smut-infected stem tips of sugarcane plants.

**Figure 5:**
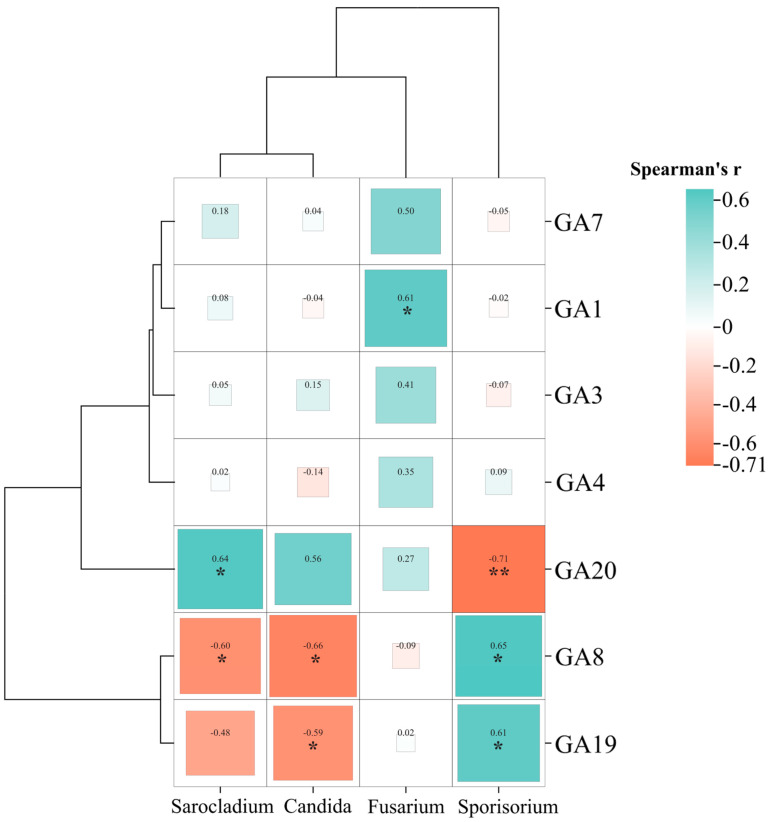
Heatmap based on Spearman’s correlation in combination with cluster analysis among selected fungal genera and GA concentrations across all samples. The sizes of the squares corresponds to the magnitudes of the values, which are also displayed within each cell. (* = *p* < 0.05; ** = *p* < 0.01).

The heatmap analysis of GA concentrations with selected fungal genera revealed significant correlations, supporting the previous results (Figure 5).

GA_19_ and GA_8_ were positively correlated with *Sporisorium*, the smut pathogen, and significantly downregulated in long stems (LS vs. SS). These GAs were negatively correlated with *Candida* and *Sarocladium*, abundant in long stem (LS) samples. Additionally, GA_20_ was highest in long-stem samples, showing a positive correlation with *Sarocladium*, followed by *Candida* and *Fusarium*, and a negative correlation with *Sporisorium*. *Fusarium* displayed a distinct trend compared with other dominant genera, being the only genus significantly positively correlated with GA_1_, GA_7_, GA3, and GA_4_ (Figure 5). These GAs were most concentrated in long whips (LWs), followed by long stems. GA_4_ and GA_7_ were exclusively present in long samples, with the highest levels in long whips. GA_1_ and GA_4_ were linked to all mentioned pathways and specifically annotated to the plant hormone signal transduction pathway. These findings highlight the significant association between endophytic fungi and gibberellin regulation and their impacts on plant physiology.

## 3. Discussion

The present study employed a combination of conventional isolation techniques and microbial community analysis to examine the fungal population diversity and the regulation of gibberellin (GA) concentrations in smut-infected sugarcanes with long and short whips. The results indicated that long shoot and whip samples (LS, LW) exhibited remarkably greater concentrations of GA_1_, GA_3_, GA_4_, and GA_7_, with the greatest concentrations detected in long whips (LW). Moreover, *Fusarium* was the only abundant genus found in whip samples associated with these GAs (GA_1_, GA_3_, GA_4_, and GA_7_). Additionally, *Candida* was associated with GA_20_, both of which were prevalent in long shoots. Therefore, it can be concluded that the presence of *Fusarium* in long whips was related to the production of GA_1_, GA_3_, GA_4_, and GA_7_. GA_1_ and GA_4_, which were involved in all four annotated pathways, and might have contributed to whip elongation through hormone signal transduction. GA_4_ and GA_7_ were not detected in short whip or stem samples.

Previous research has revealed that gibberellin promotes plant height in sugarcane through stem elongation [23]. A separate study reported that *Pseudomonas aeruginosa* (Gessard) B18-infected sugarcane plants producing IAA, GA_3_, ABA, and ETH coped with better tolerance to smut pathogen stress [24]. The present study observed greater gibberellic acid (GA_3_) production in long shoot and long whip samples (LS; LW). However, several studies have revealed that various microbes interacting with plants also produce GAs, which do not regulate host development, but promote infection by inhibiting immunity [25,26]. These findings support our results regarding the higher gibberellin concentration in long-whip samples. Through community abundance analysis, conventional isolation techniques, and UPLC-MS/MS analysis, we investigated the fungal communities and gibberellin hormones associated with smut whip length elongation in silico.

The present research employed community abundance analysis to identify significant variations in the fungal communities between shoot and whip samples. These variations can be attributed to abiotic factors, multiple taxa, and the host plant, potentially leading to endophyte-mediated plant features in the long run [27,28]. The prominent fungal genera identified in shoot samples (LS; SS) comprised *Candida*, *Sporisorium*, *Fusarium, Hanseniaspora*, and *Papiliotrema*. Meanwhile, in whip samples (LW; SW), the dominant genera were *Sporisorium*, *Candida*, and *Fusarium* (>1%). In whip samples, *Fusarium* emerged as the most prominent genus after *Sporisorium*, exclusively in long whips, while *Candida* was more prevalent in short whips. Notably, *Fusarium* species were detected in all samples except for short whips, an absence that warrants further investigation in future research focused on short whips. Yeast genera, i.e., *Candida, Hanseniaspora*, and *Papiliotrema*, accounted for 41.84% of the total fungal diversity in shoot samples, which aligns with the previous findings [29], with *Candida* being significantly abundant (57.94%) in long-stem (LS) samples. *Candida* species are renowned for their capacity to promote plant development, degrade organic contaminants, and exhibit significant aminocyclopropane-1-carboxylate deaminase (ACCD) activity, which acts as an ethylene suppressor and a growth promoter for plants [30,31,32,33]. In addition to the community abundance analysis, cultivable endophytic fungi were isolated from the samples using the conventional isolation technique and identified through ITS amplification. Consistent with the present study’s findings, several *Fusarium* species, including *F. fujikuroi*, *F. proliferatum*, and *F. verticillioides*, have previously been identified in smut-infected sugarcane whips [34,35,36]. *F. concentricum*, a member of the *F. fujikuroi* species complex (FFPC), has been isolated from rice infected with bakanae disease [37].

Gibberellins, particularly GA_3_, have been proven to stimulate cell division and elongation at the cellular level [38]. Moreover, the fungus *Sporisorium* infects plants by undergoing mitotic division of its sori within the infected meristem region [39]. KEGG enrichment analysis identified six DAMs (GA_20_, GA_8_, GA_3_, GA_1_, GA_4_, and GA_7_) in both LS vs. SS and LW vs. SW comparison analyses. All these DAMs, except for GA_20_ (highest in long stems), had their highest concentrations in long whips and were linked to the diterpenoid biosynthesis pathway. GA_8_ was exclusively annotated to this pathway and positively associated with *Sporisorium*. Terpenoids are specialized metabolites essential for direct defense against biotic and abiotic stresses. Similarly, many monoterpenes possess fungicidal and insecticidal properties [40]. Therefore, the detection of these DAMs, with the highest concentrations in long whips, along with their role in upregulated diterpenoid biosynthesis, suggests that, after the smut pathogen infiltrated the apical meristem region, the plant’s internal defense mechanisms were activated. This response likely involved pathogen-associated molecular pattern (PAMP)-triggered immunity (PTI), which could subsequently be suppressed by pathogen-induced effector-triggered susceptibility (ETS) [41].

The higher abundance of the endophytic fungus *Candida* in both long and short shoots (LSs; SSs) and its positive correlation with GA_20_ suggests that it could be involved in the stimulation of stem growth in sugarcane samples, particularly in long shoots (LSs), which exhibited the highest concentration of GA_20_. GA_20_ is a bioactive gibberellin (GA_1_, GA_3_) precursor that promotes cell elongation and increases shoot height [42,43]. A previous study demonstrated that *Candida tropicalis* HY produced plant growth regulators and stimulated growth in rice seedlings [30]. *Sporisorium*, the causal agent of smut [8], was found in all samples, with higher concentrations observed in whip samples (LW; SW). Previous studies have revealed that *Sporisorium* spp. (*S. reilianum*) stimulates inflorescence and inhibits apical dominance in maize by down-regulating the GA20-oxidase gene. This finding is consistent with our results, suggesting that *S. scitamineum* is somewhat involved in the down-regulation of GA_20_ in short whips (SW), having the lowest GA_20_ content, leading to altered plant morphology [14].

Several *Fusarium* species are potentially hazardous as they can produce toxins that affect pasture fodder and field agricultural products [44]. *Fusarium* species also produce a wide range of bioactive secondary metabolites associated with their biosynthetic genes [45]. For nearly a century, the isolate *F. fujikuroi* MP-C, part of the *Gibberella fujikuroi* (Sawada) species complex, has been recognized for the industrial-scale production of gibberellic acid [15,46,47]. Moreover, significant GA production has been reported in strains *F. proliferatum* (orchid-strain N1), *F. proliferatum* (KGL0401), and *F. konzum* MP-I (Zeller, Summerell & J.F. Leslie) [46,48,49]. In this study, secondary metabolite biosynthesis was annotated with five DAMs, among which four showed a significant correlation with *Fusarium*. After *Sporisorium*, *Fusarium* was exclusively detected in long whips (LW), which also exhibited the highest concentrations of GA_3_, GA_1_, GA_4_, and GA_7_, among which GA_1_ is available in the developing parts of plants, including shoots, leaves, and flowers [50,51], while GA_4_ is a highly growth-promoting hormone [52,53] that acts as a precursor of GA_1_, GA_7_, and GA_3_ production [54]. These findings suggest that *Fusarium* may influence GA regulation in long whips, promoting terpene biosynthesis and upregulating plant hormone signaling pathways, which lead to whip elongation. It is also possible that other endophytes contribute to this process.

Consequently, it can be inferred that the association of GAs (GA_1_, GA_7_, and GA_4_) with *Fusarium* actively stimulated hormone signal transduction within the shoot meristem area, leading to the elongation of sori and hence, longer whip lengths. These findings provide valuable insights into the role of endophytic fungi in gibberellin hormone regulation and their impact on smut-infected sugarcanes.

## 4. Materials and Methods

### 4.1. Sampling Site and Experiment Design

**Figure 6:**
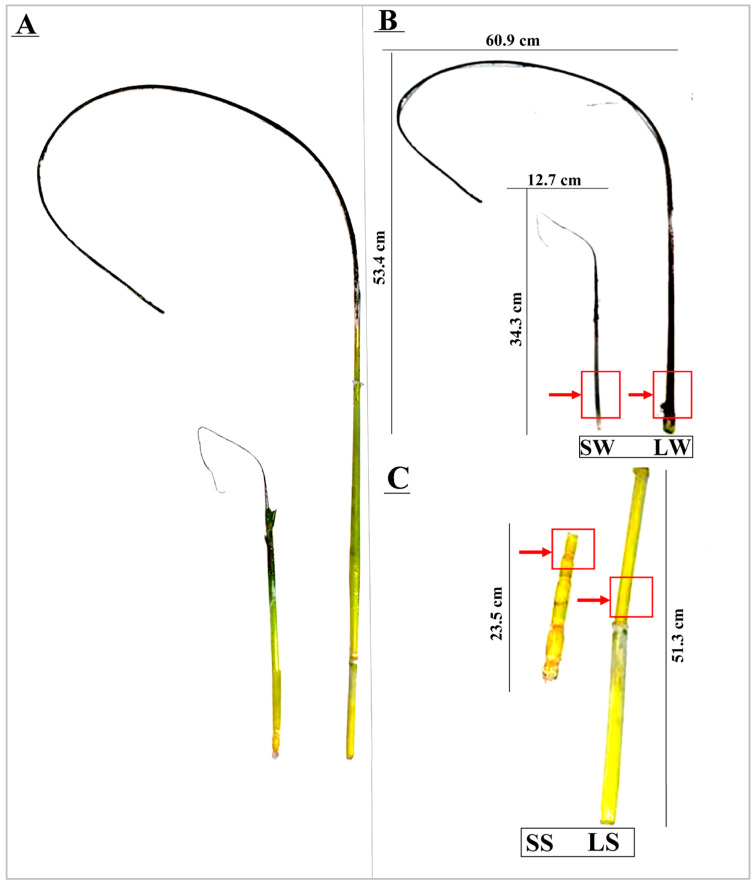
Samples of smut-infected sugarcane stalks with whips of different lengths. (**A**) Collected sample (intact); (**B**) Separated long and short whip samples; (**C**) Separated long and short stalks of corresponding whip samples. Red blocks and arrows indicate the part utilized for conventional fungal isolation from shoot samples. LW: Long whip; SW: Short whip; LS: Long stem; SS: Short stem.

This experiment was conducted from 2021 to 2022, with samples collected from the highly smut-susceptible sugarcane cultivar ROC22, planted at the Guangxi University Field Station in Fusui (22°38′06″ N, 107°54′15″ E), China. Sugarcane stems displaying distinct long and short whips were harvested and shade-dried, followed by meticulous separation of whips from stems, yielding four sample types: LWs (long whips, >100 cm), SWs (short whips, <50 cm), LSs (long stalks corresponding to long whips), and SSs (short stalks corresponding to short whips) (Figure 6). Both metagenomic and targeted metabolomic analyses were conducted using six biological replicates per sample. All samples were stored at 4 °C until further use.

### 4.2. Conventional Isolation of Endophytic Fungi

Potato dextrose agar (PDA; Sigma-Aldrich, St. Louis, MO, USA) was used as the growth medium (Table S2). For fungal isolation from stalk samples, 2 mm stem disks were disinfected with ethanol (30 s) and sterile water (30 s, twice), air-dried, and placed on PDA plates. For whips, smut spores were collected and suspended in 0.01% Tween-20/ddH_2_O (*v*/*v*, 1:100). A 100 µL suspension was spread on PDA plates [55,56]. Plates were incubated at 28 °C and monitored daily for fungal growth. Distinct colonies were transferred to fresh PDA plates, and single spore cultures were established [57]. The purity of the cultures was validated by microscopic investigation (Figure S1).

### 4.3. DNA Isolation and ITS Region Amplification

Genomic DNA from isolated fungal samples was extracted using the Cetyltrimethyl ammonium bromide (CTAB) method [58]. For metagenomic DNA (mDNA), plant samples were crushed in liquid nitrogen and processed with the QIAAmp^®^ DNA Mini Kit (Qiagen, Shanghai, China). DNA quality was measured with a NanoDrop 2000 (Thermo Fisher Scientific, Shanghai, China). The extracted DNA was used to amplify the ITS1-ITS2 gene region with universal primers ITS1F (5′-TCCGTAGGTGAACCTGCGG-3′) and ITS4R (5′-TCCTCCGCTTATTGATATGC-3′). For metagenomic samples, barcoded ITS1F/ITS4R primers were synthesized. PCR was performed using an ABI GeneAmp Model 9700 thermocycler (details in Tables S3 and S4). *Sporisorium scitamineum* (S201301 and S201302) from Fujian, China, was used as a positive control. Amplicons were subjected to electrophoresis on 2% agarose gel.

### 4.4. ITS Sequencing of Isolated Fungi

Amplified genomic DNA products were sent to Shanghai Biotech (CN) for ITS sequencing. Sequences were identified using NCBI’s BLAST with a 99% similarity threshold. From each set of identical samples, one strain was chosen based on optimal growth rate and nucleotide identity. This method allowed for the selection of diverse strains and insights into the most frequently isolated strains (Figures S1 and S2).

### 4.5. Illumina Sequencing for Metagenomic Analysis

mDNA PCR products were detected and quantified using the QuantiFluor-ST assay (Promega, Beijing, China). DNA libraries were created with the Illumina TruSeq DNA sample preparation kit and sequenced on the Illumina HiSeq 2500 platform, producing 270 bp paired-end reads (Gene Denovo Biotechnology Co., Ltd., Guangzhou, China). Raw sequences were assembled into clean reads using Fast Length Adjustment of Short reads (FLASH v1.2.11) [59] software filtering out reads < 20 bp, those with <20 quality in a 10 bp window, and those containing N bases. Paired-end reads with a 10 bp overlap and a maximum mismatch ratio of 0.2 were merged and de novo assembly was conducted using Uparse (version 7.0.1090 http://drive5.com/uparse/, assessed on 10 January 2024). The most abundant sequence in each cluster was selected as the representative sequence. Operational taxonomic units (OTUs) were assigned using QIIME (Quantitative Insights into Microbial Ecology v.1.9.0) software with the UCLUST algorithm and the Greengene database at a 97% similarity threshold. Sequences were mapped to representative sequences, yielding valid sequences for each OTU based on a minimum count of two and 8% prevalence. The OTU table was generated, and the relative abundance of different taxa was calculated for each sample. All sequencing and OTU generation analyses were performed using the Majorbio Cloud Platform (CN; https://cloud.majorbio.com/, assessed on 2 February 2024).

### 4.6. Alpha and Beta Diversity Analyses

The Majorbio Cloud Platform (CN; https://cloud.majorbio.com/, assessed on 2 February 2024) was used for further analysis of detected OTUs. Alpha diversity (Chao, Shannon, and Simpson indices) and beta diversity (principal coordinate analysis; PCoA) were analyzed to investigate OTU richness and diversity. Chao, Shannon, and Simpson indices were calculated using Mothur software (version v.1.30.2, https://mothur.org/wiki/calculators/, assessed on 2 February 2024). Principal coordinate analysis (PCoA) with the Anosim test and Euclidean algorithm was performed using R software (version 3.3.1, R Foundation for Statistical Computing, Vienna, Austria) [60]. Venn diagrams of shared OTUs, community richness bar plots, and pie plots were also generated using R software (version 3.3.1) [60].

### 4.7. Metabolomic Analysis of Targeted Metabolites

MetWare Biotechnology Co., Ltd. (http://www.metware.cn/; Wuhan, China, assessed on 6 February 2024) conducted the analysis employing their proprietary MS2 spectral tag library (MWDB, Wuhan Meiwei Biotechnology Co., Ltd., Wuhan, China) and public databases for metabolite information. The gibberellin (GAs) concentrations in sugarcane samples (stem: LS, SS; whip: LW, SW) were quantified by UHPLC-MS/MS. Preparation: 50 g of ground sample was mixed with 10 μL of isotope-labeled internal standard (conc. 100 ng/mL) and 500 μL of acetonitrile/water (ACN/H_2_O; 90:10), vortexed, and centrifuged (4 °C, 12,000 r/min, 10 min). The collected supernatants were treated with 10 µL of BPTAB and 10 µL of TEA, incubated (1 h, 90 °C), air-dried, redissolved in ACN/H_2_O (100 µL; 90:10), filtered, and analyzed via UHPLC-MS/MS (UPLC, ExionLC™ AD, /https://sciex.com.cn/, assessed on 6 February 2024), and GA quantification was performed using the AB Sciex QTRAP 6500 LC-MS/MS platform. Details of the UPLC and LC-MS/MS apparatus are given in Table S5. Scheduled MRM was performed for gibberellins, quantified via Multiquant 3.0.3. The mass spectrometer parameters were optimized with declustering potentials (DPs) and collision energies (CEs). Analysis was controlled by Analyst 1.6.3. The absolute Log2FC (fold change) values were calculated to identify significantly regulated metabolites between groups.

### 4.8. KEGG Annotation and Enrichment Analysis

Metabolites with fold changes above 2.0 or below 0.5 (VIP ≥ 1) were identified as differentially accumulated metabolites (DAMs). Identified metabolites were annotated using the KEGG compound database (http://www.kegg.jp/kegg/compound/, assessed on 21 February 2024), and differentially annotated metabolites (DAMs) were then mapped to the KEGG Pathway database (http://www.kegg.jp/kegg/pathway.html/, assessed on 21 February 2024). Pathways mapped with significantly regulated metabolites were then fed into MSEA (metabolite sets enrichment analysis), and their significance was determined by the hypergeometric test’s *p*-values [61].

## 5. Conclusions

In conclusion, this study provides new insights into the fungal dynamics and gibberellin production associated with sugarcane smut. Our analyses revealed a diverse fungal community within the whips of smut-infected sugarcanes, with *Sporisorium* and *Fusarium* genera being prominent, especially in longer whips. Conventional isolation also identified several *Fusarium* isolates from whip samples. UHPLC-MS/MS identified higher levels of gibberellin hormones (GA_1_, GA_4_, GA_7_, and GA_3_) in these longer whips, with *Fusarium* showing a strong positive correlation with these GAs. KEGG enrichment analysis further linked these hormones to diterpenoid biosynthesis and plant hormone signal transduction pathways. These results suggest that *Fusarium* may significantly contribute to the production of GAs (GA_1_, GA_4_, GA_7_, and GA_3_) and the elongation of sugarcane whips. Other detected endophytes may also contribute to this process. This study lays the groundwork for future investigations into the molecular mechanisms underlying gibberellin synthesis and its regulation in the context of sugarcane smut.

## Figures and Tables

**Table 1:** Alpha diversity indices of long and short smut-infected sugarcane stem and whip samples.

Sample\Estimators	Chao	Shannon	Simpson
Long stem (LS)	58.95	1.66	0.34
Short stem (SS)	103.88	2.31	0.19
Long whip (LW)	38.05	0.25	0.90
Short whip (SW)	38.92	0.38	0.84

**Table 2:** Endophytic fungi isolated from smut-infected sugarcane stalk and whip samples of varying lengths following PCR amplification and 99% nucleotide similarity results.

No.	Isolated Fungi Code	PCR Amplicon Number	Type of Sugarcane	Identified Species	Plant Part
**1**	A	2	Long	*Mucor irregularis*	Stem
**2**	B	4	Long	*Fusarium proliferatum*	Stem
**3**	C	5	Long	*Fusarium pseudocircinatum*	whip
**4**	D	6	Long	*Fusarium* sp. DBF13KW4b	Stem
**5**	E	7	Long	*Daldinia eschscholtzii*	Stem
**6**	F	8	Long	*Epicoccum sorghinum*	Whip
**7**	G	9	Long	*Mucor irregularis*	Whip
**8**	H	10	Long	*Fusarium fujikuroi*	Whip
**9**	I	11	Long	*Fusarium* sp. ASR-126	Whip
**10**	J	12	Long	*Fusarium verticillioides*	Whip
**11**	K	13	Short	*Fusarium* sp. ASR-126	Whip
**12**	L	14	Short	*Fusarium fujikuroi*	Whip
**13**	M	15	Short	*Fusarium* sp. ASR-126	Stem
**14**	N	16	Short	*Fusarium temperatum*	Stem
**15**	O	17	Short	*Fusarium fujikuroi*	Stem
**16**	P	18	Short	*Fusarium proliferatum*	Stem
**17**	Q	19	Short	*Sarocladium* sp. BAB-5555	Stem
**18**	R	20	**Short**	***Sporisorium scitamineum***	Whip
**19**	S	21	Short	*Mucoromycotina* sp.	Stem
**20**	T	22	Short	*Fusarium chlamydosporum*	Whip
**21**	U	23	**Positive Control**	***Sporisorium scitamineum***	--

**Table 3:** Gibberellin (GA) contents (ng/g) in different smut-infected sugarcane stalk and whip samples.

Metabolite	Long Stem (LS)	Short Stem (SS)	Short Whip (SW)	Long Whip (LW)
**GA20**	0.81 ± 0.48 **a**	0.14 ± 0.07 **a**	0.03 ± 0.02 **a**	0.18 ± 0.10 **a**
**GA1**	2.58 ± 1.59 **b**	1.05 ± 0.14 **b**	0.72 ± 0.19 **b**	10.24 ± 6.96 **a**
**GA3**	10.10 ± 9.24 **b**	0.49 ± 0.69 **c**	0.65 ± 0.92 **c**	34.01 ± 46.38 **a**
**GA8**	0.00	0.19 ± 0.27	0.37 ± 0.37 **b**	1.77 ± 1.70 **a**
**GA19**	0.00	0.54 ± 0.77	1.51 ± 0.39 **a**	1.42 ± 1.02 **a**
**GA4**	0.003 ± 0.01	0.00	0.00	0.40 ± 0.50
**GA7**	0.10 ± 0.14	0.00	0.00	0.54 ± 0.70

Different letters indicate significant differences between different concentrations of the same sample (*p* ≤ 0.05).

## Data Availability

The data supporting this study’s findings are all provided along with this manuscript.

## Supplementary Materials

The following supporting information can be downloaded at: https://www.mdpi.com/article/10.3390/ijms25169129/s1.
