# Supplementary figures and images for: Fungal Diversity and Gibberellin Hormones Associated with Long Whips of Smut-Infected Sugarcanes
Source: Int J Mol Sci. 2024 Aug 22;25(16):9129. doi: 10.3390/ijms25169129 (PMC11355029; doi:10.3390/ijms25169129)

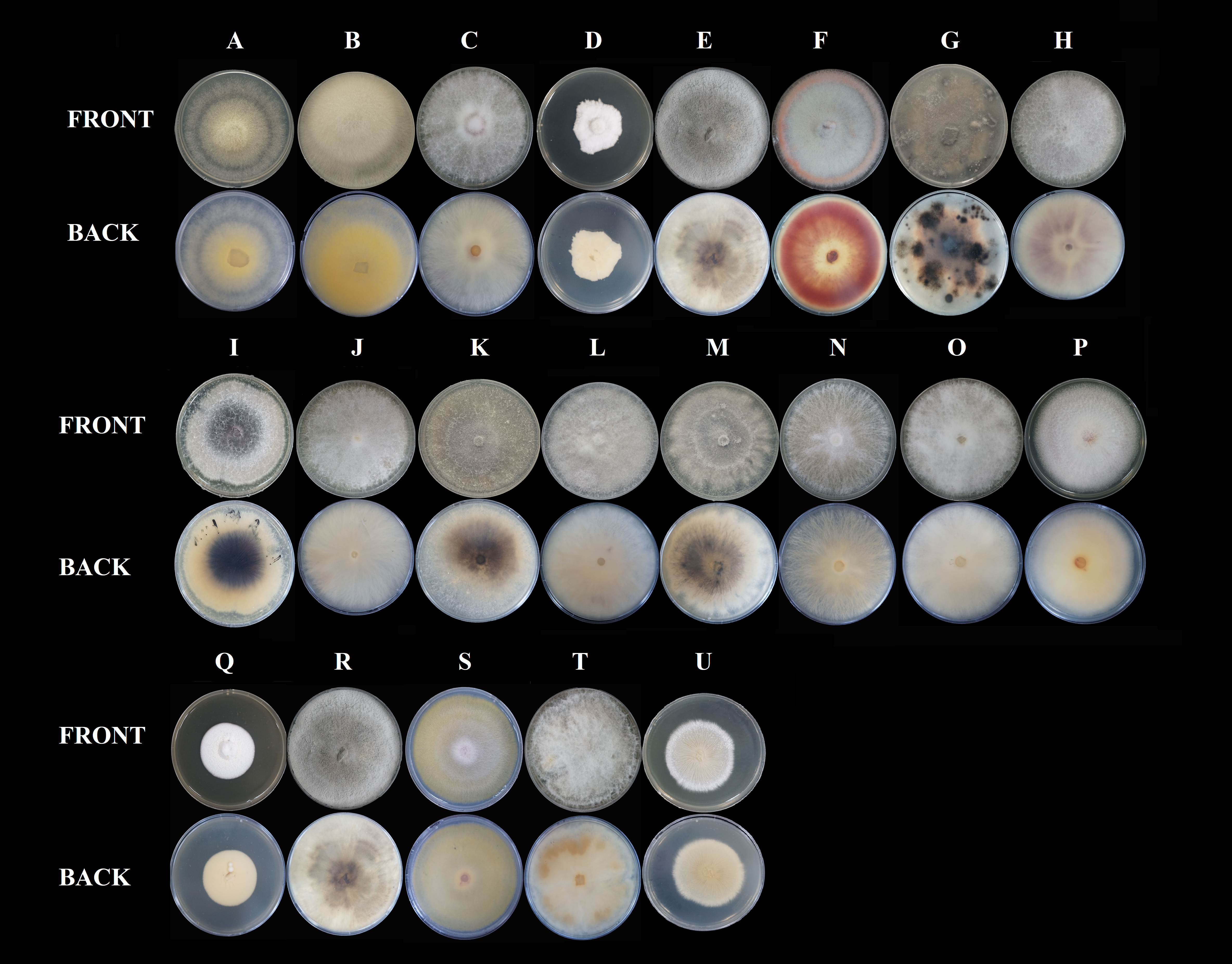

Supplement: Supplementary file 1 [file ijms-25-09129-s001.zip › Figure S1.tif]

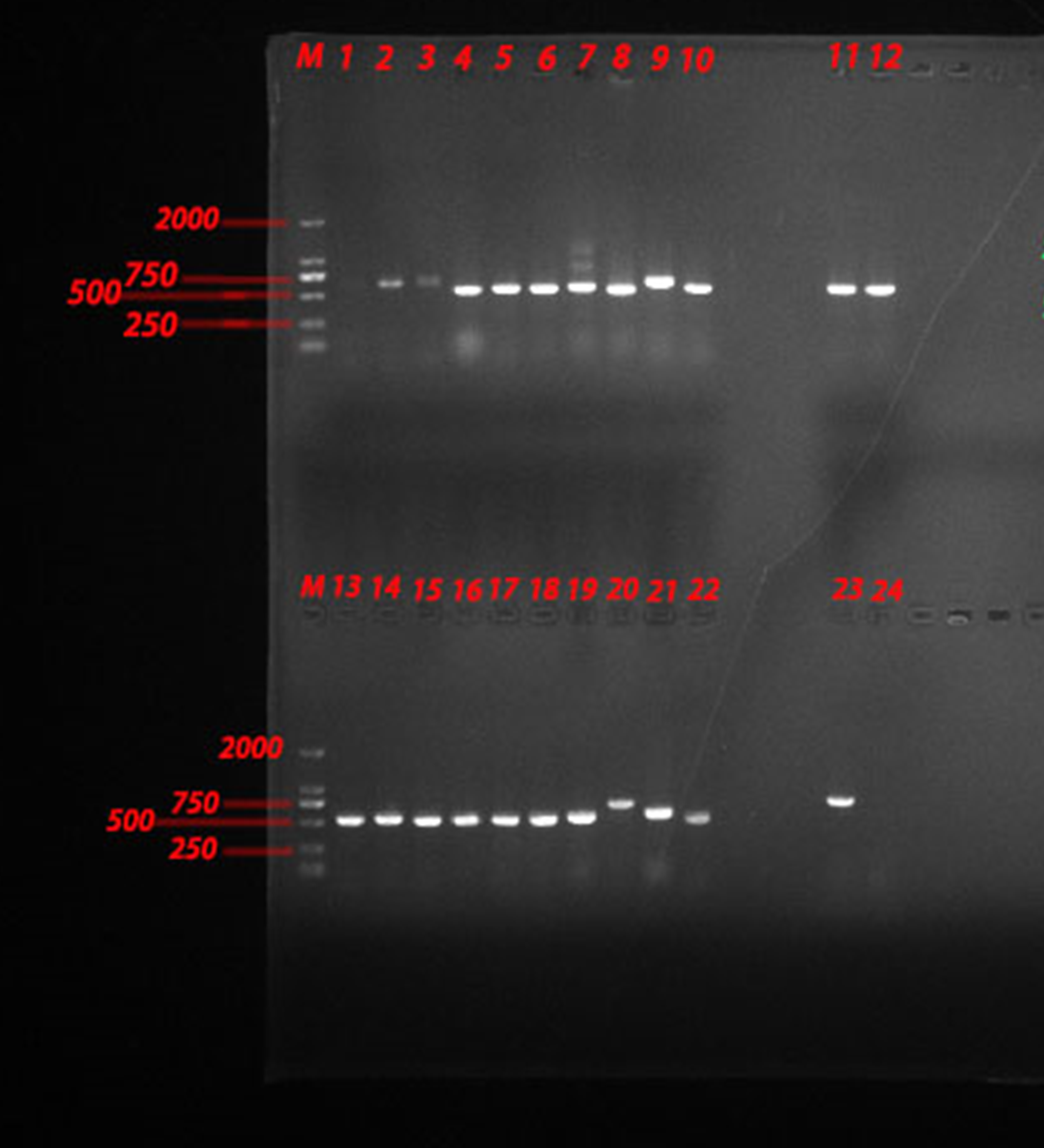

Supplement: Supplementary file 1 [file ijms-25-09129-s001.zip › Figure S2.tif]
